# *DTX3* copy number increase in breast cancer: a study of associations to molecular subtype, proliferation and prognosis

**DOI:** 10.1007/s10549-021-06138-2

**Published:** 2021-02-22

**Authors:** Marit Valla, Signe Opdahl, Borgny Ytterhus, Anna Mary Bofin

**Affiliations:** 1 Department of Clinical and Molecular Medicine, Faculty of Medicine and Health Sciences, Norwegian University of Science and Technology, 7491 Trondheim, Norway; 2 Department of Pathology, St. Olav’s Hospital, Trondheim University Hospital, 7006 Trondheim, Norway; 3 Department of Public Health and Nursing, Faculty of Medicine and Health Sciences, Norwegian University of Science and Technology, 7491 Trondheim, Norway

**Keywords:** DTX3, Breast cancer, Copy number increase, Proliferation, Prognosis

## Abstract

**Purpose:**

The degree of cell proliferation is important for subclassification of breast cancers into prognostic and therapeutic groups. DTX3 has been identified as a driver of proliferation in luminal breast cancer. In this study, we describe DTX3 copy number in breast cancer primary tumours and corresponding axillary lymph node metastases, and studied associations with molecular subtype, proliferation and prognosis.

**Methods:**

Using fluorescence in situ hybridization, we assessed DTX3 and chromosome 12 centromere (CEP12) copy number in 542 primary breast cancers and 117 lymph node metastases, from a well-described cohort of Norwegian breast cancer patients. Proliferation was expressed as mitotic counts and Ki67 score. Associations between DTX3 copy number and molecular subtype and proliferation were assessed using Pearson’s *χ*^2^ test. We studied the effect of copy number increase on prognosis estimating cumulative incidence of breast cancer death and hazard ratios.

**Results:**

Mean DTX3 copy number ≥ 4 was found in 23 tumours (4%), and mean ≥ 5 in 9 tumours (1.7%). Copy number increase was found within all molecular subtypes except the 5 negative phenotype and the Luminal B (HER2 +) subtype. DTX3 copy number increase was not accompanied by an increase in CEP12. Point estimates showed that there were associations between DTX3 copy number increase and high proliferation and poor prognosis; however, precision depended on copy number cut-off.

**Conclusions:**

DTX3 copy number increase was present in a small proportion of breast cancer cases. There was an association between copy number increase and high tumour cell proliferation and poor prognosis.

## Introduction

Members of the Deltex (DTX) family have ubiquitin-protein isopeptide ligase activity [1], can regulate transcription [2], and are involved in neurogenesis and the Notch signalling pathway [2–4]. *DTX3,* one of the genes included in the DTX family, is located on the long arm of chromosome 12 (12q13.3) [5]. In a recent study of oesophageal cancer, *DTX3* was found to increase degradation of NOTCH2 and reduce proliferation and migration of oesophageal cancer cells [4]. It was thus identified as an anti-oncogene in oesophageal cancer and a potential target for therapy [4].

Proliferation is one of the hallmarks of cancer [6, 7], and the degree of proliferation is important for subclassification of breast cancer into the intrinsic molecular subtypes [8, 9]. Molecular subtyping can also be done using immunohistochemistry (IHC) and in situ hybridization (ISH) as surrogates for gene expression analyses [10–13]. In many countries, the proliferation marker Ki67 is used to subdivide luminal breast cancers into prognostic and therapeutic groups, but its use as a predictive biomarker is debated.

Given the key role of proliferation in breast cancer subclassification, prognostication, and management, identification of new proliferation-associated genes could be important. A previous study has shown that *DTX3* is a driver of proliferation in luminal (non-basal) breast cancer [14]. Furthermore, *DTX3* amplification was associated with poor prognosis in this subgroup of breast cancer patients. Luminal tumours comprise a large proportion of all breast cancers, and identification of new prognostic markers within this heterogeneous group could prove to be of clinical use.

In this study, we used fluorescence in situ hybridization (FISH) on a historic, well-characterized cohort of Norwegian breast cancer patients to characterize *DTX3* copy number in 542 formalin-fixed, paraffin-embedded primary breast cancers and their corresponding lymph node metastases. We aimed to assess a possible association between *DTX3* copy number status in the primary tumours, and molecular subtype, proliferation and prognosis. Furthermore, we studied whether there was an association between *DTX3* copy number status in the primary tumours and in the corresponding lymph node metastases.

## Material and methods

### Patient characteristics

The cohort comprises 25,727 women from Nord-Trøndelag County, Norway, born between 1886 and 1928. They were invited to attend a clinical screening for the early clinical detection of breast cancer between 1956 and 1959 [15]. Information regarding incident breast cancers was obtained from the Cancer Registry of Norway. For follow-up data, the Norwegian cause of death registry [16] was used. The women were followed for breast cancer occurrence between 1961 and 2008, and during this period, 1379 new breast cancers were registered. Of these, 909 were reclassified into molecular subtypes and described in a previous study [10]. From the time of diagnosis, patients were followed until death from breast cancer, death from other causes, or until 31 December 2015, whichever came first. Individual information regarding adjuvant treatment is unavailable for the study cohort. However, according to Norwegian guidelines at the time of diagnosis, none of the patients would have received targeted anti-HER2 treatment. Due to high patient age at diagnosis and the time of diagnosis, few would have qualified for antihormonal treatment and/or adjuvant chemotherapy.

### Specimen characteristics

The primary tumours were previously reclassified into histological type and grade [10, 17]. Tissue microarray (TMA) blocks were made using the Tissue Arrayer Mini-Core with TMA Designer2 software (Alphelys). From the periphery of FFPE primary tumours and from the lymph node metastases, three tissue cores (diameter 1 mm) were transferred to TMA recipient blocks. TMA Sections (4 μm) were cut and mounted on Superfrost + glass slides, dried overnight at 37 °C and stored in the freezer at − 20 °C. All primary tumours were reclassified into molecular subtypes [10]. The molecular subtypes were defined as follows: Luminal A (oestrogen receptor (ER) and/or progesterone receptor (PR)^+^, Human epidermal growth factor receptor 2 (HER2)^−^, Ki67 < 15%), Luminal B (HER2^−^) (ER^+^ and/or PR^+^, HER2^−^, Ki67 ≥ 15%), Luminal B (HER2^+^) (ER^+^ and/or PR^+^, HER2^+^), HER2 type (ER^−^ and PR^−^, HER2^+^), 5 negative phenotype (5NP; ER^−^, PR^−^, HER2^−^, Cytokeratin 5 (CK5)^−^ and Epidermal growth factor receptor (EGFR)^−^) and Basal phenotype (BP; ER^−^, PR^−^, HER2^−^, CK5^+^ and/or EGFR^+^). ER, PR, Ki67, CK5 and EGFR status was determined by IHC. HER2 status was determined by chromogenic in situ hybridization (CISH) or IHC [10].

**Fig. 1:**
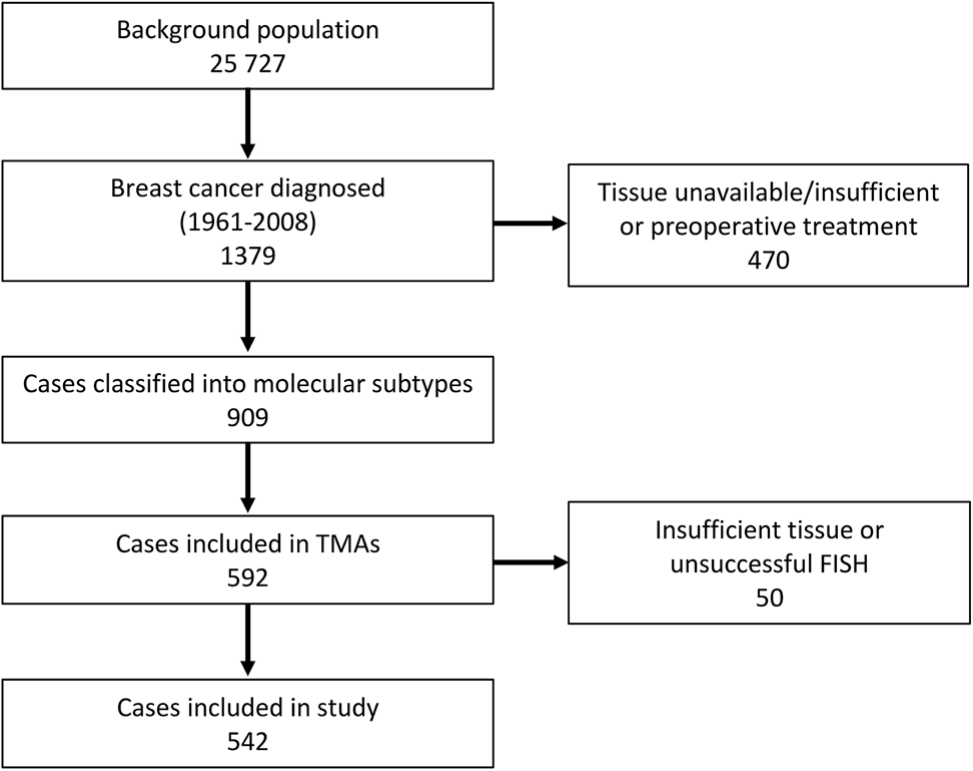
Overview of study population

In the present study, only TMAs containing cores from tumours diagnosed mainly in the 1980s or later were included (*n* = 592) (Fig. 1). Of these, 37 were excluded due to unsuccessful FISH and 13 were excluded due to insufficient amounts of tumour tissue. Thus, 542 cases were suitable for *DTX3* and chromosome enumeration probe 12 (CEP12) copy number assessment. Of these, 181 had lymph node metastases, and lymph node tissue from 124 cases was included in TMAs. Cases with unsuccessful FISH (*n* = 4) or insufficient amounts of tumour tissue (*n* = 3) were excluded. Hence, lymph node metastases from 117 cases were included.

FISH was done according to the manufacturer’s guidelines, using Dako Histology FISH Accessory Kit K 579911. TMA slides were de-waxed and rehydrated, and then boiled in a microwave oven for 10 min in Pre-Treatment Solution. The slides were cooled for 15 min, washed in Wash Buffer (2 × 3 min.), and protein digested with Pepsin Solution at 37 °C for 30 min. Slides were then washed in Wash buffer (2 × 3 min.). Dehydration was done in ethanol for 2 min at each concentration (70, 80 and 95%), and the slides were air dried for 15 min at room temperature. *DTX3* (3 μL, Empire Genomics) and CEP12 (1 μL, Abbott/VYSIS) FISH-custom probes were mixed with hybridization buffer (9 μL, Empire Genomics) and applied to the TMA slides. Slides were coverslipped and sealed with coverslip sealant (Dako). Denaturation was done at 83 °C for 3 min, and hybridization was done in a DAKO Hybridizer at 37 °C overnight. After hybridization, TMA slides were rinsed in 0.4xSSC/0.3%NP-40 at 72 °C for 2 min, in 2xSSC/0.1%NP-40 at room temperature for 15 s and air dried at 37 °C for 15 min. DAPI (15 μL, VYSIS. Abbott no. 06J50-001) was applied and the slides were coverslipped.

### Scoring and reporting

For each case, we examined all available tissue cylinders using a fluorescence microscope (Nikon Eclipse 90i). We recorded *DTX3* and CEP12 copy numbers in 20 non-overlapping and well-preserved tumour cell nuclei. For each case, we estimated mean *DTX3* and mean CEP12 copy number per tumour cell. There are no established guidelines for subclassification of cases based on mean *DTX3* copy number. Based on *HER2* guidelines [18], the cases were initially separated into three categories: mean *DTX3* copy number < 4; mean ≥ 4 < 6; and mean ≥ 6. The same subclassification was used for CEP12 copy number. Due to few cases with mean *DTX3* ≥ 6 (*n* = 3), we also separated the cases into two groups for the prognostic studies; mean *DTX3* copy number < 4 versus mean ≥ 4, and mean *DTX3* copy number < 5 versus mean ≥ 5. The REMARK criteria for tumour marker reporting were followed [19].

### Statistical analyses

We used Pearson chi-square tests to compare *DTX3* copy number status in the primary tumours across patient and tumour characteristics. Paired analysis (McNemar’s test) was used to compare copy number status in the primary tumours and the corresponding lymph node metastases.

We estimated cumulative incidence of death from breast cancer according to mean *DTX3* copy number (mean < 4 versus mean ≥ 4 and mean < 5 versus mean ≥ 5) in the prognostic analyses. We defined death from other causes as a competing event and used Gray’s test to compare equality of the cumulative incidence curves. For assessment of relative risk, we used Cox proportional hazard models to estimate hazard ratios (HR) of breast cancer death with 95% confidence intervals (CI), censoring at time of death from other causes. Adjustments were made for the following covariates in separate models: age at diagnosis (≤ 49, 50–59, 60–64, 65–69, 70–74, ≥ 75), stage (I–IV), histological grade (I–III), and Ki67 status (< / ≥ 15%). There were no clear violations of proportionality in log-minus-log plots.

All statistical tests were two-sided, and statistical significance was defined at 5% level. *p*-values between 5 and 10% were considered borderline significant. We used STATA version 15.1 (Stata Corp., College Station, TX, USA) in the statistical analyses.

## Results

Mean age at diagnosis was 75.3 years, and mean follow-up after diagnosis was 9.1 years (Table 1). Mean age at diagnosis was lower among cases with *DTX3* copy number increase, compared to cases with low *DTX3* copy number.

**Table 1:** Patient and tumour characteristics

	Total study population	Mean *DTX3* copy number				Mean *DTX3* copy number			Mean *DTX3* copy number		
		< 4	≥ 4 < 6	≥ 6	*p* value (*χ*^2^)	< 4	≥ 4	*p* value (*χ*^2^)	< 5	≥ 5	*p* value (*χ*^2^)
*N* (%)	542	519 (95.8)	20 (3.7)	3 (0.6)		519 (95.8)	23 (4.2)		533 (98.3)	9 (1.7)	
Mean age at diagnosis, years (SD)	75.3 (8.4)	75.4 (8.2)	72.8 (9.6)	71.0 (23.4)		75.4 (8.2)	72.6 (11.4)		75.4 (8.3)	68.6 (12.6)	
Mean follow-up, years (SD)	9.1 (7.2)	9.1 (7.2)	9.3 (9.3)	3.4 (1.6)		9.1 (7.2)	8.5 (8.9)		9.1 (7.3)	5.9 (5.5)	
Deaths from breast cancer (%)	191 (35.2)	180 (34.7)	9 (45.0)	2 (66.7)		180 (34.7)	11 (47.8)		184 (34.5)	7 (77.8)	
Deaths from other causes (%)	297 (54.8)	287 (55.3)	9 (45.0)	1 (33.3)		287 (55.3)	10 (43.5)		295 (55.4)	2 (22.2)	
Histologic grade (%)											
I	60 (11.0)	60 (11.6)	0 (0)	0 (0)	< 0.001	60 (11.6)	0	< 0.001	60 (11.3)	0	0.3
II	305 (56.3)	299 (57.6)	6 (30)	0 (0)		299 (57.6)	6 (26.1)		301 (56.5)	4 (44.4)	
III	177 (32.7)	160 (30.8)	14 (70)	3 (100)		160 (30.8)	17 (73.9)		172 (32.3)	5 (55.6)	
Lymph node metastasis (%)											
Yes	181 (33.4)	172 (33.1)	7 (35.0)	2 (66.7)	0.7	172 (33.1)	9 (39.1)	0.7	177 (43.2)	3 (42.9)	0.5
No	236 (43.5)	226 (43.6)	9 (45.0)	1 (33.3)		226 (43.6)	10 (43.5)		233 (56.8)	4 (57.1)	
Unknown histology	125 (23.1)	121 (23.3)	4 (20.0)	0 (0)		121 (23.3)	4 (17.4)		123 (23.1)	2 (22.2)	
Tumour size (%)											
≤ 2 cm	263 (48.5)	255 (49.1)	6 (30.0)	2 (66.7)	0.09	255 (49.1)	8 (34.8)	0.07	260 (48.8)	3 (33.3)	0.7
> 2 ≤ 5 cm	95 (17.5)	86 (16.6)	9 (45.0)	0 (0)		86 (16.6)	9 (39.1)		93 (17.5)	2 (22.2)	
> 5 cm	10 (1.9)	10 (1.9)	0 (0)	0 (0)		10 (1.9)	0		10 (1.9)	0	
Uncertain, but > 2 cm	64 (11.8)	60 (11.6)	3 (15)	1 (33.3)		60 (11.6)	4 (17.4)		62 (11.6)	2 (22.2)	
Uncertain	110 (20.3)	108 (20.8)	2 (10.0)	0 (0)		108 (20.8)	2 (8.7)		108 (20.3)	2 (22.2)	
Stage (%)											
I	261 (48.2)	252 (48.6)	8 (40.0)	1 (33.3)	0.6	252 (48.6)	9 (39.1)	0.3	256 (48.0)	5 (55.6)	0.8
II	226 (41.7)	212 (40.9)	12 (60.0)	2 (66.7)		212 (40.9)	14 (60.9)		222 (41.7)	4 (44.4)	
III	29 (5.3)	29 (5.6)	0 (0)	0 (0)		29 (5.6)	0		29 (5.4)	0	
IV	24 (4.4)	24 (4.6)	0 (0)	0 (0)		24 (4.6)	0		24 (4.5)	0	
Unknown	2 (0.3)	2 (0.4)	0 (0)	0 (0)		2 (0.4)	0		2 (0.4)	0	
Molecular subtype (%)											
Luminal A	288 (53.1)	280 (54.0)	7 (35.0)	1 (33.3)	0.2	280 (54.0)	8 (34.8)	0.2	285 (53.5)	3 (33.3)	0.003
Luminal B (HER2-)	130 (24.0)	122 (23.5)	8 (40.0)	0 (0)		122 (23.5)	8 (34.8)		129 (24.2)	1 (11.1)	
Luminal B (HER2 +)	43 (7.9)	42 (8.1)	1 (5)	0 (0)		42 (8.1)	1 (4.4)		43 (8.1)	0	
HER2 type	30 (5.5)	27 (5.2)	2 (10.0)	1 (33.3)		27 (5.2)	3 (13.0)		27 (5.1)	3 (33.3)	
5NP	12 (2.2)	12 (2.3)	0 (0)	0 (0)		12 (2.3)	0		12 (2.3)	0	
BP	39 (7.2)	36 (6.9)	2 (10.0)	1 (33.3)		36 (6.9)	3 (13.0)		37 (6.9)	2 (22.2)	
Histological subtype (%)											
Ductal carcinoma	377 (69.6)	358 (69.0)	16 (80.0)	3 (100)	1.0	358 (69.0)	19 (82.6)	0.8	370 (69.4)	7 (77.8)	0.7
Lobular carcinoma	68 (12.6)	67 (12.9)	1 (5.0)	0 (0)		67 (12.9)	1 (4.4)		67 (12.6)	1 (11.1)	
Tubular carcinoma	1 (0.2)	1 (0.2)	0 (0)	0 (0)		1 (0.2)	0		1 (0.2)	0	
Mucinous carcinoma	28 (5.2)	28 (5.4)	0 (0)	0 (0)		28 (5.4)	0		28 (5.3)	0	
Medullary carcinoma	13 (2.4)	12 (2.3)	1 (5.0)	0 (0)		12 (2.3)	1 (4.4)		12 (2.3)	1 (11.1)	
Papillary carcinoma	26 (4.8)	25 (4.8)	1 (5.0)	0 (0)		25 (4.8)	1 (4.4)		26 (4.9)	0	
Metaplastic	8 (1.5)	8 (1.5)	0 (0)	0 (0)		8 (1.5)	0		8 (1.5)	0	
Other	21 (3.9)	20 (3.9)	1 (5.0)	0 (0)		20 (3.9)	1 (4.4)		21 (3.9)	0	
Ki67 high/low (%)											
Ki67 < 15%	327 (60.3)	318 (61.3)	8 (40.0)	1 (33.3)	0.1	318 (61.3)	9 (39.1)	0.03	323 (60.6)	4 (44.4)	0.3
Ki67 ≥ 15%	215 (39.7)	201 (38.7)	12 (60.0)	2 (66.7)		201 (38.7)	14 (60.9)		210 (39.4)	5 (55.6)	
Mitoses/10 HPF, median (IQR p25, p75)	5 (1, 12)	5 (1, 11)	12 (6.5, 23.5)	12 (9, 43)		5 (1, 11)	12 (8, 25)		5 (1, 12)	10 (9, 14)	
Mitoses/10 HPF, quartiles (%)											
≤ 1	143 (26.4)	141 (27.2)	2 (10.0)	0 (0)	0.03	141 (27.2)	2 (8.7)	0.009	142 (26.6)	1 (11.1)	0.3
> 1, ≤ 5	146 (26.9)	143 (27.6)	3 (15.0)	0 (0)		143 (27.6)	3 (13.0)		145 (27.2)	1 (11.1)	
> 5, ≤ 12	130 (24.0)	123 (23.7)	5 (25.0)	2 (66.7)		123 (23.7)	7 (30.4)		126 (23.6)	4 (44.4)	
> 12	123 (22.7)	112 (21.6)	10 (50.0)	1 (33.3)		112 (21.6)	11 (47.8)		120 (22.5)	3 (33.3)	

*SD* standard deviation, *HER2* human epidermal growth factor receptor 2, *5NP* 5 negative phenotype, *BP* basal phenotype, *HPF* high power fields

### *DTX3* in primary tumours and association with molecular subtype, proliferation and histological grade

**Fig. 2:**
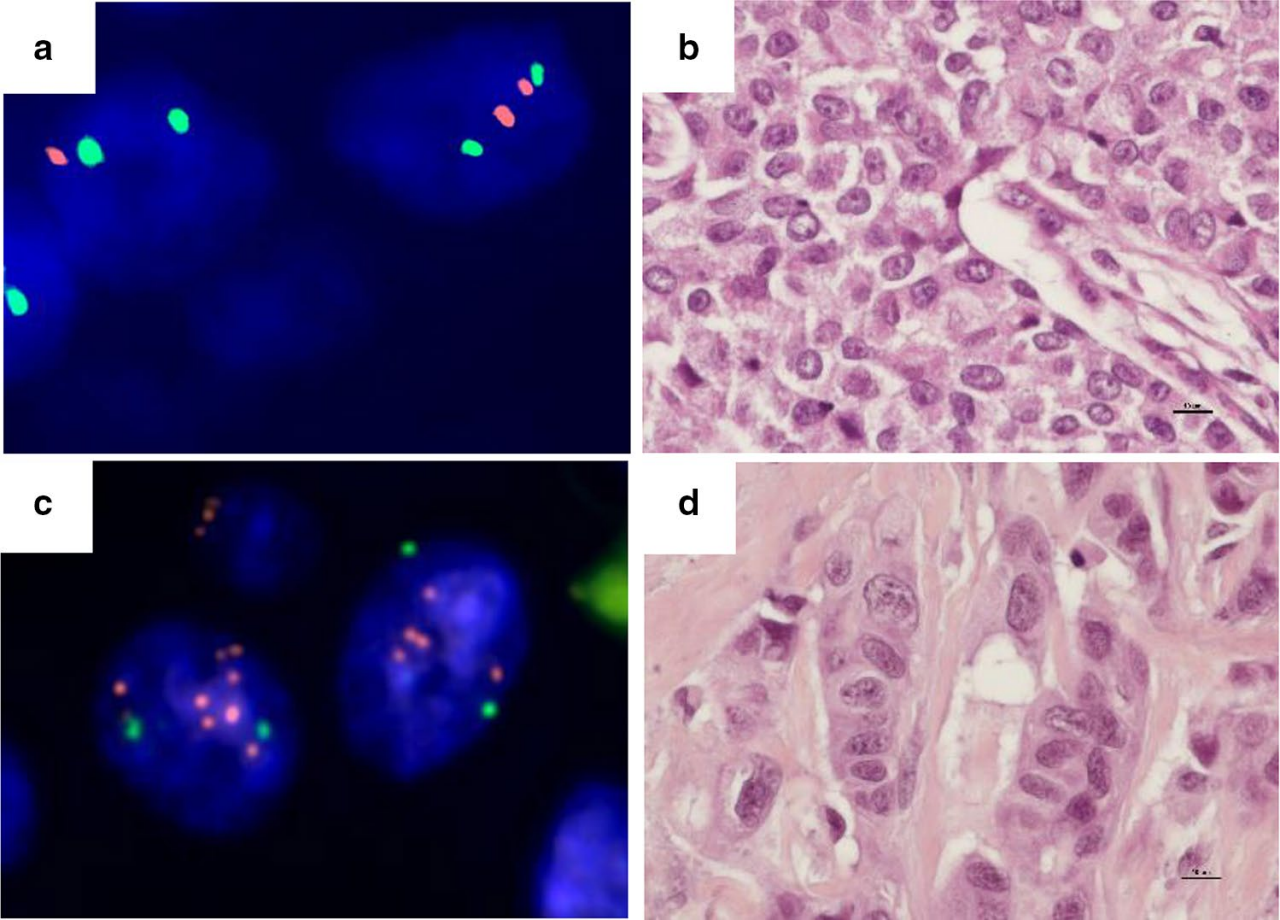
**a** Breast cancer cell nuclei without copy number increase of *DTX3* (red) and CEP12 (green). **b** Hematoxylin-erythrosine-saffron (HES)-stained section (× 600) of breast cancer tumour shown in a. Scale bar = 10 µm. **c** Breast cancer cell nuclei with copy number increase of *DTX3* (red), and two copies of CEP12 (green). **d** HES-stained section (× 600) of breast cancer tumour shown in c. Scale bar = 10 µm

Mean *DTX3* copy number ≥ 4 < 6 was found in the primary tumours of 20 (3.7%) cases, and mean copy number ≥ 6 was found in only three (0.6%) cases. Mean *DTX3* copy number ≥ 5 was found in the primary tumours of nine (1.7%) cases (Table 1, Fig. 2). Mean copy number ≥ 4 was found within all molecular subtypes except the 5NP, and mean copy number ≥ 5 was found within all molecular subtypes except Luminal B (HER2^+^) and the 5NP. Mean copy number ≥ 6 was found in Luminal A, HER2 type and BP.

Point estimates showed that Ki67 levels were higher among cases with *DTX3* copy number increase, compared to cases with low *DTX3* copy number, regardless of cut-off for mean *DTX3* copy number (Table 1). However, a statistically significant association between *DTX3* copy number increase and Ki67 levels was only found using the < 4/ ≥ 4 cut-off. Point estimates also showed that mitotic counts were higher among cases with *DTX3* copy number increase, regardless of cut-off. Statistically significant associations between *DTX3* copy number increase and mitotic counts were found using the < 4/ ≥ 4 < 6/ ≥ 6 and the < 4/ ≥ 4 cut-offs. Furthermore, point estimates showed that a higher proportion of cases with *DTX3* copy number increase had histological grade III tumours, compared to cases with low copy number. Statistically significant associations between *DTX3* copy number increase and histological grade were found using the < 4/ ≥ 4 < 6/ ≥ 6 and the < 4/ ≥ 4 cut-offs.

### *DTX3* and CEP12

**Fig. 3:**
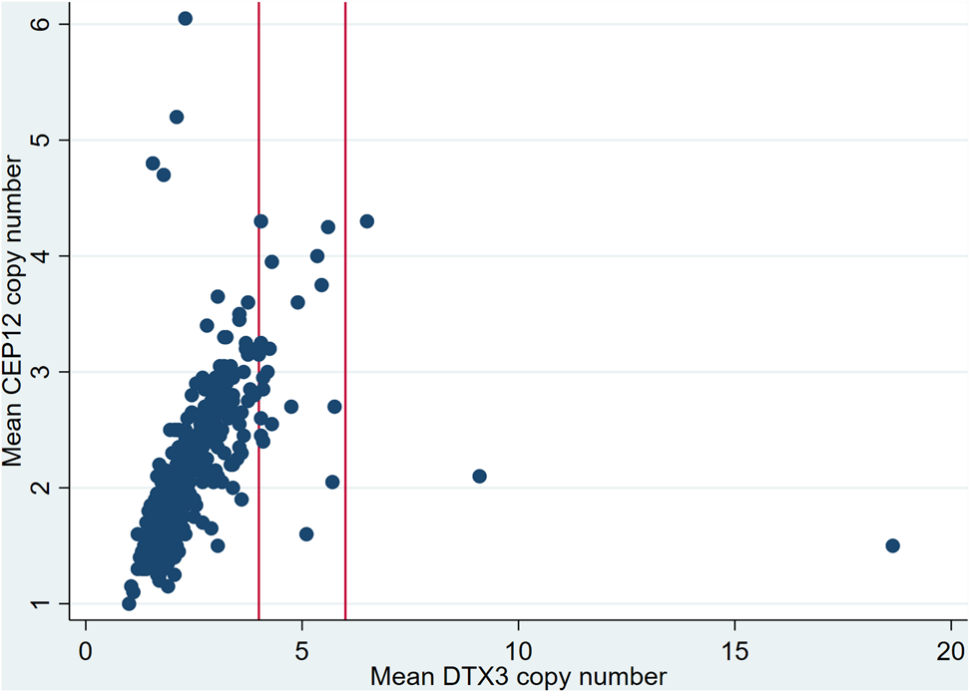
Scatterplot of *DTX3* and CEP12 copy numbers. Red vertical lines indicate *DTX3* copy number 4 (left) and 6 (right)

CEP12 copy number was infrequent in our study population; only seven cases (1.3%) had mean CEP12 ≥ 4 < 6 and one case (0.2%) had mean ≥ 6 (Table 2, Fig. 3). Of the 23 cases with mean *DTX3* copy number ≥ 4, only four cases had a concurrent increase in CEP12 copy number.

**Table 2:** *DTX3* and CEP12 copy number in primary tumours

	Mean *DTX3* copy number (%)				
	< 4	≥ 4 < 6	≥ 6	Total	*χ*^2^ test
Mean CEP12 copy number (%)					
< 4	515 (99.2)	17 (85.0)	2 (66.7)	534	*p* < 0.0001
≥ 4 < 6	3 (0.6)	3 (15.0)	1 (33.3)	7	
≥ 6	1 (0.2)	0	0	1	
Total	519	20	3	542

### *DTX3* in lymph node metastases

Only one case with mean *DTX3* ≥ 6 in the primary tumour had available tissue from the corresponding lymph node metastasis. For this case, mean *DTX3* ≥ 6 was also found in the lymph node metastasis (Table 3). Among the six cases with mean *DTX3* copy number ≥ 4 < 6 in the primary tumours, three (50%) also had mean ≥ 4 < 6 in the lymph node metastases.

**Table 3:** *DTX3* status in primary tumours and lymph node metastases

	Mean *DTX3* copy number in primary tumours			
	< 4	≥ 4 < 6	≥ 6	Total
Mean *DTX3* copy number in lymph nodes (%)				
< 4	108 (98.2)	3 (50.0)	0 (0)	111
≥ 4 < 6	2 (1.8)	3 (50.0)	0 (0)	5
≥ 6	0 (0)	0 (0)	1 (100)	1
Total	110 (100)	6 (100)	1 (100)	117

	Mean *DTX3* copy number in primary tumours^a^			
	< 4	≥ 4		Total
Mean *DTX3* copy number in lymph nodes (%)				
< 4	108 (98.2)	3 (42.9)		111
≥ 4	2 (1.8)	4 (57.1)		6
Total	110 (100)	7 (100)		117

	Mean *DTX3* copy number in primary tumours^b^			
	< 5	≥ 5		Total
Mean *DTX3* copy number in lymph nodes (%)				
< 5	114 (99.1)	1 (50.0)		115
≥ 5	1 (0.9)	1 (50.0)		2
Total	115 (100)	2 (100)		117

^a^McNemar test: *p* = 0.65

^b^McNemar test: *p* = 1.0

### *DTX3* and prognosis

**Fig. 4:**
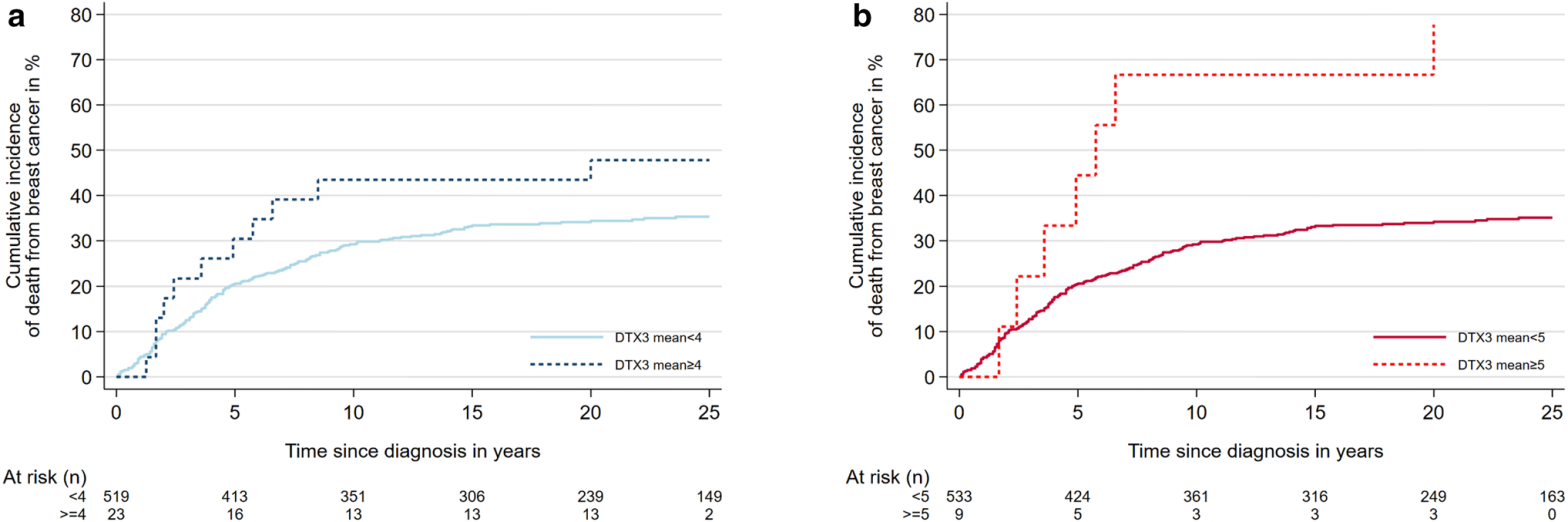
Cumulative incidence of breast cancer death according to *DTX3* copy number status. **a** Mean *DTX3* < 4 versus mean ≥ 4 (Gray’s test: *p* = 0.17). **b** Mean *DTX3* < 5 versus mean ≥ 5 (Gray’s test: *p* = 0.003)

Ten years after diagnosis, the cumulative risk of death from breast cancer for cases with mean *DTX3* copy number < 4 was 29% (95% CI 26–33) (Table 4; Fig. 4a). For cases with mean copy number ≥ 4, the corresponding risk was 44% (95% CI 26–66). There were no clear differences in the rates of death between the two categories in the Cox regression analysis, although point estimates were higher for cases with mean copy number ≥ 4. Adjusting for age, stage, grade and Ki67 status had no clear impact on the results.

**Table 4:** Absolute and relative risk of death from breast cancer according to *DTX3* status

	Mean *DTX3* copy number	
	< 4	≥ 4
Number of cases (%)	519 (95.8)	23 (4.2)
Cumulative risk after 5 years (%) (95% CI)	20.4 (17.2–24.2)	30.4 (15.8–53.4)
Cumulative risk after 10 years (%) (95% CI)	29.2 (25.5–33.3)	43.5 (26.2–65.7)
HR, unadjusted (95% CI)	1.0	1.51 (0.82–2.78)
HR, adjusted for age (95% CI)	1.0	1.47 (0.79–2.74)
HR, adjusted for stage (95% CI)	1.0	1.63 (0.88–3.03)
HR, adjusted for grade (95% CI)	1.0	1.13 (0.60–2.12)
HR, adjusted for Ki67 (95% CI)	1.0	1.23 (0.67–2.29)

	Mean *DTX3* copy number	
	< 5	≥ 5
Number of cases (%)	533 (98.3)	9 (1.7)
Cumulative risk after 5 years (%) (95% CI)	20.5 (17.3–24.1)	44.4 (19.6–79.6)
Cumulative risk after 10 years (%) (95% CI)	29.2 (25.5–33.3)	66.7 (37.7–92.2)
HR, unadjusted (95% CI)	1.0	3.13 (1.47–6.68)
HR, adjusted for age (95% CI)	1.0	3.35 (1.55–7.26)
HR, adjusted for stage (95% CI)	1.0	4.37 (2.03–9.39)
HR, adjusted for grade (95% CI)	1.0	2.82 (1.32–6.03)
HR, adjusted for Ki67 (95% CI)	1.0	3.07 (1.44–6.57)

*CI* confidence interval, *HR* hazard ratio

The cumulative risk of death from breast cancer for cases with mean *DTX3* copy number < 5 was 29% (95% CI 26–33) ten years after diagnosis (Table 4; Fig. 4b). For cases with mean copy number ≥ 5, the corresponding risk was 67% (95% CI 38–92). In the Cox regression analyses, the rate of death from breast cancer was higher among cases with mean *DTX3* copy number ≥ 5, compared to mean < 5 (HR 3.1 (95% CI 1.5–6.7)). Adjusting for age, stage, grade and Ki67 status had no clear impact on the results.

## Discussion

We identified *DTX3* copy number increase in a low proportion of breast cancer cases (4.2% of cases had mean *DTX3* ≥ 4, and 1.7% of cases had mean *DTX3* ≥ 5). It was seen in all molecular subtypes except the 5NP and the Luminal B (HER2^+^). Point estimates showed that cases with *DTX3* copy number increase had higher proliferation, higher histologic grade and a poorer prognosis than cases without copy number increase. However, whether the associations were statistically significant depended on the choice of cut-off.

In this study, we initially subdivided tumours into three categories based on mean *DTX3* copy number: < 4; ≥ 4 < 6; and ≥ 6. Since only three patients had tumours with mean *DTX3* copy number ≥ 6, we subdivided patients into two groups (Mean *DTX3* < 4 versus mean ≥ 4; and mean *DTX3* < 5 versus mean ≥ 5) in the prognostic analyses. In the study by Gatza et al. [14], *DTX* amplification was identified in 5% of tumours in the METABRIC dataset, and in 18% in the TCGA dataset. Thus, the frequency of tumours with copy number increase in our cohort was more similar to the METABRIC dataset, than to the TCGA dataset. Mean age at diagnosis was high in our study cohort (75 years), compared to the METABRIC (mean age 61 years) and the TCGA (mean age 58 years) datasets. In our cohort, mean age at diagnosis was lower among cases with *DTX3* copy number increase compared to cases with low *DTX3* (72.6 versus 75.4 years using the < 4/ ≥ 4 cut-off). To elucidate whether the low proportion of *DTX3* amplified tumours found in our study population could be associated with high age, it would be interesting to study *DTX3* copy number using an in situ method such as FISH in a young cohort of breast cancer patients. In the study by Gatza et al*.,* amplifications were identified through SNP-based copy number analysis, whereas in our study, copy number assessment was done by FISH.

We identified mean *DTX3* copy number ≥ 4 in all molecular subtypes except the 5NP, and mean *DTX3* copy number ≥ 5 in all molecular subtypes except the 5NP and Luminal B (HER2^+^). Gatza et al. however, found that amplifications were restricted to highly proliferative luminal tumours. In our study, molecular subtyping was based on surrogate markers, and luminal tumours were defined as all tumours that were ER and/or PR positive [10]. In Gatza et al. molecular subtyping was done by gene expression analysis, and luminal tumours were defined as all tumours that were not basal [14, 20]. The definition of “luminal” was therefore different in the two studies. We found *DTX3* copy number ≥ 4 in eight Luminal A cases. These tumours are by definition not considered highly proliferative [8, 10]. However, of these eight tumours, five were histological grade III and could possibly represent misclassified Luminal B tumours [12, 21]. Using the < 5/ ≥ 5 cut-off, high copy number was found in three Luminal A cases. Of these, one was histological grade III. Similar to Gatza et al. we found an association between *DTX3* copy number increase and proliferation. We assessed proliferation through Ki67 levels and mitotic counts, whereas Gatza et al. used the PAM50 proliferation signature [9].

Gatza et al. found that *DTX3* amplification was associated with a poor prognosis [14]. In our study, regardless of cut-offs for *DTX3* copy number, point estimates showed that patients with *DTX3* copy number increase had higher risks of death from breast cancer compared to cases without copy number increase. However, due to few cases within the high copy number groups, and wide confidence intervals, the results regarding prognosis must be interpreted with caution. By defining low copy number increase as mean copy number ≥ 4 and ≥ 5, we may have concealed some of the prognostic effect of true *DTX3* amplification. When subdividing cases into three categories (mean < 4; ≥ 4 < 6; ≥ 6), there was a higher rate of death among cases with mean *DTX3* ≥ 6 in the Cox regression analysis (HR 3.9, 95% CI 1.0–15.8). However, only three cases had mean ≥ 6, and using a higher cut-off was not feasible for our cohort.

In breast cancer, there is an association between high tumour proliferation and a poor prognosis [22, 23]. The prognostic effect of proliferation has been shown to be age dependent, with a stronger prognostic influence among younger breast cancer patients [24]. Therefore, if *DTX3* exerts its influence on prognosis through proliferation, a study of the prognostic effect of *DTX3* copy number increase among younger breast cancer patients would be of interest.

*DTX3* may play different roles in different cancers. The gene was suggested as a tumour suppressor gene in oesophageal cancer [4], and it has been proposed as an endogenous control gene for gene expression analyses in colorectal cancer [25]. Further studies are needed to elucidate the role of *DTX3* copy number increase on prognosis in breast cancer.

A strength of our study is the use of a well-characterized Norwegian breast cancer cohort, with unusually long follow-up [10]. Long follow-up is important in studies of breast cancer prognosis, as breast cancer patients, especially those with hormone receptor-positive disease, may experience relapse decades after their primary diagnosis. Identification of patients included in this study and registration of follow-up data were done through linkage with reliable national registries. In Norway, reporting of cancer is regulated by law [26], and reporting to the Cancer Registry of Norway is therefore near complete [27]. With regard to our choice of methodology, using an in situ technique such as FISH ensures that only invasive breast cancer cells are assessed. Furthermore, FISH is a well-established method, and compared to modern multigene assays, it is time- and cost-effective and therefore feasible in most laboratories. Molecular subtyping of this series of tumours was done in a previous study, using the same subtyping algorithm, the same laboratory, antibodies and cut-off levels for all cases.

There are also some limitations to our study. We found few cases with *DTX3* copy number increase in the primary tumours. Confidence intervals were wide in the prognostic analyses, and the results must therefore be interpreted with caution. Precision may be influenced both by the number of cases, the number of breast cancer deaths in the study population, and the distribution of cases between the categories of *DTX3* copy number status [28]. The lack of individual information on breast cancer treatment is a limitation. Furthermore, we used TMAs for assessment of *DTX3* and CEP12 copy number. Although studies have shown good concordance between TMAs and whole sections [29, 30], intratumour heterogeneity and thus representativity may be a problem. Our TMAs were specifically selected from the periphery of the primary tumours. We observed that when *DTX3* copy number increase was present, it was seen in the majority of cancer cells in all three TMAs. Thus, copy number changes were not as focal as previously observed for another proliferation marker (*FGD5*) in the same cohort [31]. However, bearing this in mind, the findings of the present study should be validated in a series of whole sections from breast cancer.

Expression of ER and PR, and overexpression of HER2, is much more frequent than copy number increase of this proliferation-associated gene in our study population. However, in the era of personalized medicine, and with the implementation of multigene classifiers, new prognostic markers that are less frequently observed than the established breast cancer biomarkers could also prove to be of clinical relevance in the future.

In conclusion, we found that *DTX3* copy number increase was present in a small proportion of breast cancer cases. Copy number increase was seen within all molecular subtypes except the 5NP and the Luminal B (HER2^+^) subtype. Compared to cases without *DTX3* copy number increase, point estimates showed that tumour proliferation and histological grade was higher, and prognosis was poorer for patients with *DTX3* copy number increase.

## References

[1] Takeyama K, Aguiar RC, Gu L, He C, Freeman GJ, Kutok JL (2003). The BAL-binding protein BBAP and related Deltex family members exhibit ubiquitin-protein isopeptide ligase activity. J Biol Chem.

[2] Kishi N, Tang Z, Maeda Y, Hirai A, Mo R, Ito M (2001). Murine homologs of deltex define a novel gene family involved in vertebrate Notch signaling and neurogenesis. Int J Dev Neurosci.

[3] Matsuno K, Diederich RJ, Go MJ, Blaumueller CM, Artavanis-Tsakonas S (1995). Deltex acts as a positive regulator of Notch signaling through interactions with the Notch ankyrin repeats. Development.

[4] Ding XY, Hu HY, Huang KN, Wei RQ, Min J, Qi C (2020). Ubiquitination of NOTCH2 by DTX3 suppresses the proliferation and migration of human esophageal carcinoma. Cancer Sci.

[5] Genecards (2016) The Human Gene Database. https://www.genecards.org. Accessed 28 Jan 2021

[6] Hanahan D, Weinberg RA (2000). The hallmarks of cancer. Cell.

[7] Hanahan D, Weinberg RA (2011). Hallmarks of cancer: the next generation. Cell.

[8] Sorlie T, Perou CM, Tibshirani R, Aas T, Geisler S, Johnsen H (2001). Gene expression patterns of breast carcinomas distinguish tumor subclasses with clinical implications. Proc Natl Acad Sci USA.

[9] Parker JS, Mullins M, Cheang MC, Leung S, Voduc D, Vickery T (2009). Supervised risk predictor of breast cancer based on intrinsic subtypes. J Clin Oncol.

[10] Engstrom MJ, Opdahl S, Hagen AI, Romundstad PR, Akslen LA, Haugen OA (2013). Molecular subtypes, histopathological grade and survival in a historic cohort of breast cancer patients. Breast Cancer Res Treat.

[11] Valla M, Vatten LJ, Engstrom MJ, Haugen OA, Akslen LA, Bjorngaard JH (2016). Molecular subtypes of breast cancer: long-term incidence trends and prognostic differences. Cancer Epidemiol Biomark Prev.

[12] Dawood S, Hu R, Homes MD, Collins LC, Schnitt SJ, Connolly J (2011). Defining breast cancer prognosis based on molecular phenotypes: results from a large cohort study. Breast Cancer Res Treat.

[13] Carey LA, Perou CM, Livasy CA, Dressler LG, Cowan D, Conway K (2006). Race, breast cancer subtypes, and survival in the Carolina Breast Cancer Study. JAMA.

[14] Gatza ML, Silva GO, Parker JS, Fan C, Perou CM (2014). An integrated genomics approach identifies drivers of proliferation in luminal-subtype human breast cancer. Nat Genet.

[15] Kvale G, Heuch I, Eide GE (1987). A prospective study of reproductive factors and breast cancer. I. Parity. Am J Epidemiol.

[16] Norwegian Cause of Death Registry (2021) https://www.fhi.no/en/hn/health-registries/cause-of-death-registry/. Accessed 28 Jan 2021

[17] Lakhani SR, Ellis IO, Schnitt SJ, Tan PH, van de Vijver MJ (2012). WHO classification of tumours of the breast.

[18] Wolff AC, Hammond MEH, Allison KH, Harvey BE, Mangu PB, Bartlett JMS (2018). Human epidermal growth factor receptor 2 testing in breast cancer: American Society of Clinical Oncology/College of American Pathologists Clinical Practice Guideline Focused Update. J Clin Oncol.

[19] McShane LM, Altman DG, Sauerbrei W, Taube SE, Gion M, Clark GM (2006). REporting recommendations for tumor MARKer prognostic studies (REMARK). Breast Cancer Res Treat.

[20] Hoadley KA, Yau C, Wolf DM, Cherniack AD, Tamborero D, Ng S (2014). Multiplatform analysis of 12 cancer types reveals molecular classification within and across tissues of origin. Cell.

[21] Coates AS, Winer EP, Goldhirsch A, Gelber RD, Gnant M, Piccart-Gebhart M (2015). Tailoring therapies-improving the management of early breast cancer: St Gallen International Expert Consensus on the Primary Therapy of Early Breast Cancer 2015. Ann Oncol.

[22] Cheang MC, Chia SK, Voduc D, Gao D, Leung S, Snider J (2009). Ki67 index, HER2 status, and prognosis of patients with luminal B breast cancer. J Natl Cancer Inst.

[23] Wirapati P, Sotiriou C, Kunkel S, Farmer P, Pradervand S, Haibe-Kains B (2008). Meta-analysis of gene expression profiles in breast cancer: toward a unified understanding of breast cancer subtyping and prognosis signatures. Breast Cancer Res.

[24] Baak JP, van Diest PJ, Voorhorst FJ, van der Wall E, Beex LV, Vermorken JB (2007). The prognostic value of proliferation in lymph-node-negative breast cancer patients is age dependent. Eur J Cancer.

[25] Kheirelseid EA, Chang KH, Newell J, Kerin MJ, Miller N (2010). Identification of endogenous control genes for normalisation of real-time quantitative PCR data in colorectal cancer. BMC Mol Biol.

[26] FOR 2001–12–21 nr 1477: Forskrift om innsamling og behandling av helseopplysninger i Kreftregisteret (Kreftregisterforskriften) [Legal regulation of the Cancer Registry of Norway, document in Norwegian.]. https://lovdata.no/dokument/SF/forskrift/2001-12-21-1477.

[27] Larsen IK, Smastuen M, Johannesen TB, Langmark F, Parkin DM, Bray F (2009). Data quality at the Cancer Registry of Norway: an overview of comparability, completeness, validity and timeliness. Eur J Cancer.

[28] Rothman K, Greenland S, Lash T (2008). Modern epidemiology.

[29] Batistatou A, Televantou D, Bobos M, Eleftheraki AG, Kouvaras E, Chrisafi S (2013). Evaluation of current prognostic and predictive markers in breast cancer: a validation study of tissue microarrays. Anticancer Res.

[30] Camp RL, Charette LA, Rimm DL (2000). Validation of tissue microarray technology in breast carcinoma. Lab Investig.

[31] Valla M, Engstrom MJ, Ytterhus B, Hansen AK, Akslen LA, Vatten LJ (2017). FGD5 amplification in breast cancer patients is associated with tumour proliferation and a poorer prognosis. Breast Cancer Res Treat.

